# Supplementary figures and images for: Detection of serum IgG autoantibodies to FcεRIα by ELISA in patients with chronic spontaneous urticaria
Source: PLoS One. 2022 Aug 19;17(8):e0273415. doi: 10.1371/journal.pone.0273415 (PMC9390921; doi:10.1371/journal.pone.0273415)

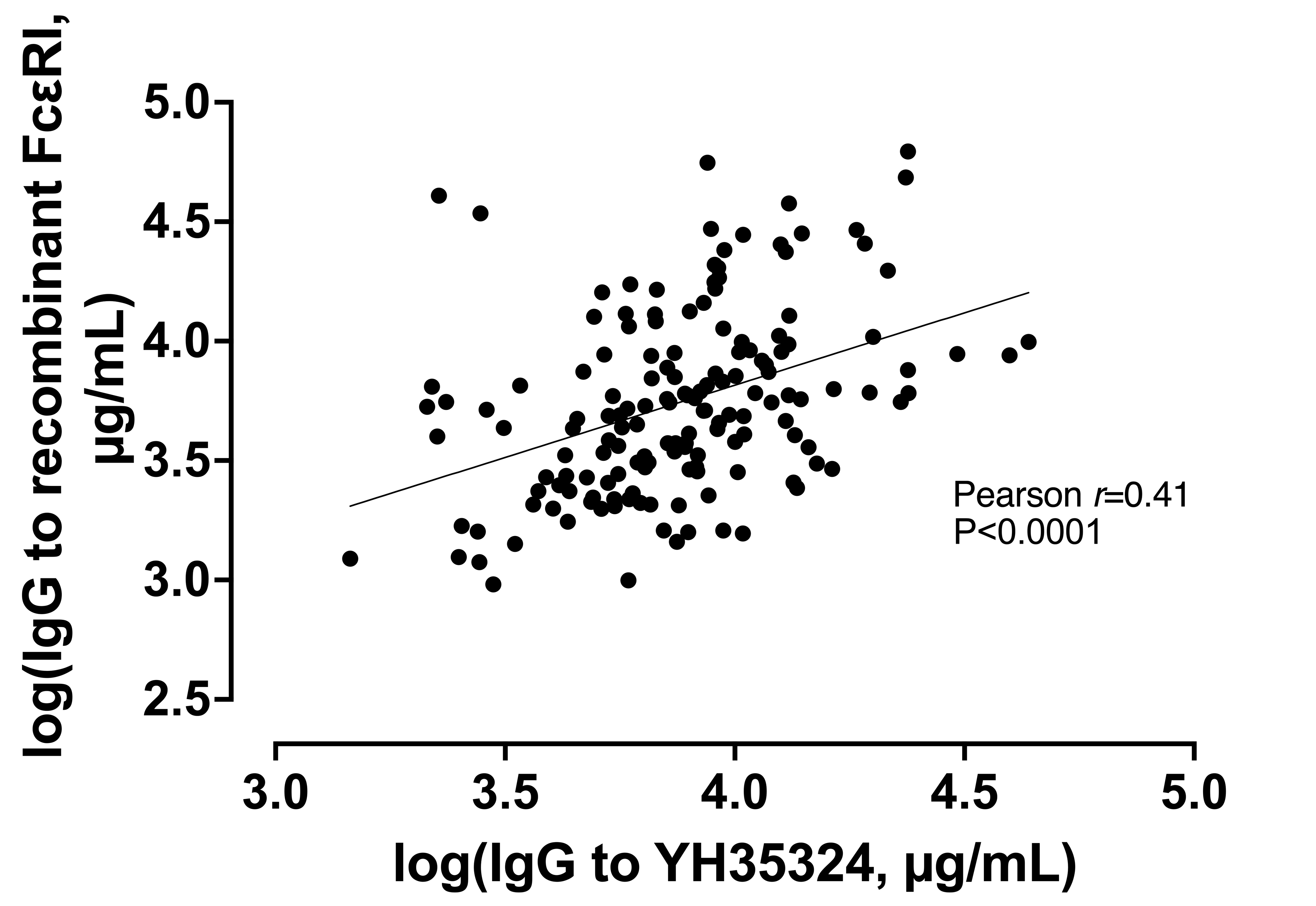

Supplement: S1 Fig — (TIF) [file pone.0273415.s001.tif]

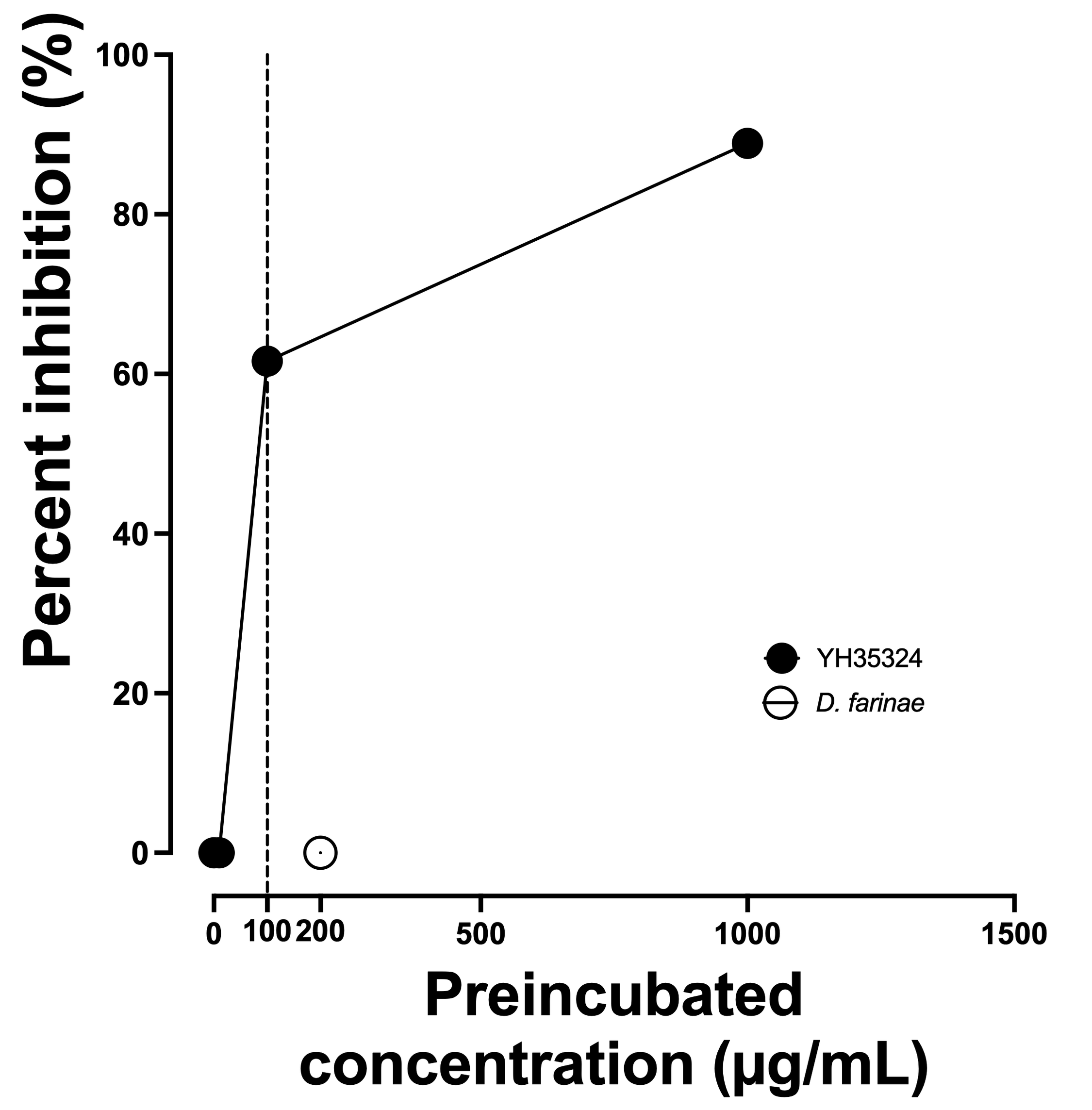

Supplement: S2 Fig — ELISA, enzyme-linked immunosorbent assay; D. farinae; Dermatophagoides farinae. (TIF) [file pone.0273415.s002.tif]

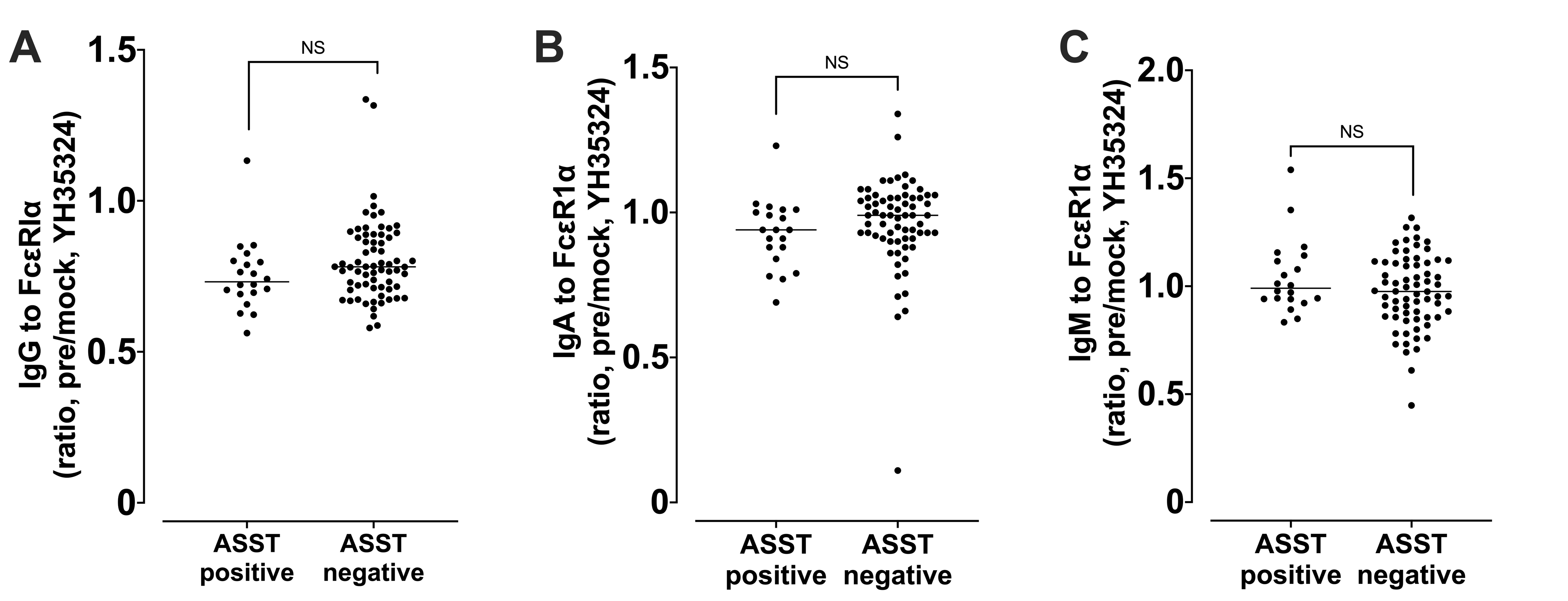

Supplement: S3 Fig — The results were presented by the IgG ratio of YH35324-pretreated to mock-treated value (A), the IgA ratio of YH35324-pretreated to mock-treated value (B) and the IgM ratio of YH35324-pretreated to mock-treated value (C). ASST, autologous serum skin test; NS, no statistical significance. (TIF) [file pone.0273415.s003.tif]
